# Non‐Obese MKR Mouse Model of Type 2 Diabetes Reveals Skeletal Alterations in Mineralization and Material Properties

**DOI:** 10.1002/jbm4.10583

**Published:** 2021-12-16

**Authors:** Matthew J.L. Tice, Stacyann Bailey, Grażyna E. Sroga, Emily J. Gallagher, Deepak Vashishth

**Affiliations:** ^1^ Department of Biomedical Engineering Center for Biotechnology and Interdisciplinary Studies, Rensselaer Polytechnic Institute New York NY USA; ^2^ Division of Endocrinology, Diabetes and Bone Diseases, Department of Medicine Icahn School of Medicine at Mount Sinai New York NY USA

**Keywords:** BONE QUALITY, GLYCATION, MINERALIZATION, NON‐OBESE, TYPE 2 DIABETES

## Abstract

Obesity is a common comorbidity of type 2 diabetes (T2D). Therefore, increased risk of fragility fractures in T2D is often confounded by the effects of obesity. This study was conducted to elucidate the mechanistic basis by which T2D alone leads to skeletal fragility. We hypothesized that obesity independent T2D would deteriorate bone's material quality by accumulating defects in the mineral matrix and undesired modifications in its organic matrix associated with increased oxidative stress and hyperglycemia. To test this hypothesis, we used 15‐week‐old male non‐obese mice with engineered muscle creatine kinase promoter/human dominant negative insulin growth factor 1 (IGF‐I) receptor (MKR) and FVB/N wild‐type (WT) controls (*n* = 12/group). MKR mice exhibit reduced insulin production and loss of glycemic control leading to diabetic hyperglycemia, verified by fasting blood glucose measurements (>250 mg/dL), without an increase in body weight. MKR mice showed a significant decrease in femoral radial geometry (cortical area, moment of inertia, cortical thickness, endosteal diameter, and periosteal diameter). Bone mineral density (BMD), as assessed by micro–computed tomography (μCT), remained unchanged; however, the quality of bone mineral was altered. In contrast to controls, MKR mice had significantly increased hydroxyapatite crystal thickness, measured by small‐angle X‐ray scattering, and elongated *c*‐axis length of the crystals evaluated by confocal Raman spectroscopy. There was an increase in changes in the organic matrix of MKR mice, associated with enhanced glycoxidation (carboxymethyl‐lysine [CML] and pentosidine) and overall glycation (fluorescent advanced glycation end products), both of which were associated with various measures of bone fragility. Moreover, increased CML formation positively correlated with elongated mineral crystal length, supporting the role of this negatively charged side chain to attract calcium ions, promote growth of hydroxyapatite, and build a physical link between mineral and collagen. Collectively, our results show, for the first time, changes in bone matrix in a non‐obese T2D model in which skeletal fragility is attributable to alterations in the mineral quality and undesired organic matrix modifications. © 2021 The Authors. *JBMR Plus* published by Wiley Periodicals LLC on behalf of American Society for Bone and Mineral Research.

## Introduction

Type 2 diabetes (T2D) is a chronic metabolic disorder characterized by reduced insulin sensitivity throughout the body contributing to hyperglycemia. As a compensatory mechanism, hyperglycemia stimulates pancreatic β‐cells to release increased insulin; however, such release becomes progressively insufficient to maintain glucose homeostasis.^(^ ^1^, ^2^ ^)^ T2D develops when defective insulin secretion is unable to compensate for the degree of insulin resistance.^(^ ^3^ ^)^

There are numerous causes of insulin resistance in humans including obesity, peroxisome proliferator–activated receptor γ (PPARγ) polymorphism, and autoimmune disease.^(^ ^2^ ^)^ In addition to causing insulin resistance, obesity is a common comorbidity in humans with T2D. Studies aiming to elucidate the mechanisms of diabetic bone fragility are confounded by the protective (eg, increased loading^(^ ^4^, ^5^ ^)^ and bone formation^(^ ^6^ ^)^) and deleterious (eg, increased inflammation^(^ ^7^ ^)^ and decreased muscle mass^(^ ^8^, ^9^ ^)^) effects of obesity on skeletal health. Obese T2D models fail to account for the nearly 11% of the diabetic population in the United States that is non‐obese.^(^ ^10^ ^)^

To this end, we selected a non‐obese murine model with transgenic expression of kinase‐dead growth factor‐I receptor under the muscle creatine kinase promoter (MKR). These mice develop dyslipidemia by the age of 2 weeks and frank diabetes with hyperglycemia by 8 weeks.^(^ ^11^, ^12^ ^)^ Thus, this model recapitulates the human diabetic condition as the progression to frank diabetes involves insulin resistance and hyperinsulinemia, with dyslipidemia.

A previous study using the MKR model identified reduced cortical bone area, caused by an increase in osteoclast number at periosteal and endosteal surfaces, with no sustained change in osteoblastic measures by 16 weeks of age.^(^ ^13^ ^)^ Bones from MKR mice displayed reduced stiffness and load to fracture. Both these changes could mostly be explained by changes in morphology and smaller cross‐sectional sizes suggesting that bone strength and modulus were not altered. The reduced cortical structure in the MKR mice is consistent with similar work done in male human T2D patients, experiencing a reduction in bone volume/total volume (BV/TV) and bone surface/bone volume (BS/BV) with no difference in bone mineral density (BMD).^(^ ^14^, ^15^ ^)^ However, MKR mice bone displayed increased postyield deformation (a measure of bone plasticity). It is noteworthy that, despite demonstrating similar changes in morphological traits and cellular activity to non‐obese T2D,^(^ ^16^ ^)^ these results of biochemical tests are in contrast to increased fracture risk seen in non‐obese human T2D condition.^(^ ^17^, ^18^ ^)^ By studying the impact of diabetes in the absence of obesity, the confounding effects of obesity on T2D would be removed. Other studies using obese diabetic males revealed the increased BMD, BV/TV, and deleterious effects of advanced glycation end products (AGEs). Thus, without the use of a non‐obese population, these results are confounded by excessive loading of the common comorbidity of obesity.

Given that skeletal changes in T2D are expected to ensue from hyperglycemia and hyperinsulinemia, we posited whether the MKR model will display enhanced bone fragility resulting from changes in bone quality rather than quantity. For example, the accumulation of AGEs, caused by hyperglycemia, hyperinsulinemia, and increased oxidative stress,^(^ ^19^ ^)^ are associated with increased bone fragility in humans with T2D. AGEs can also alter bone structure in a manner similar to obesity^(^ ^5^ ^)^ by suppressing turnover through alterations in osteoclastic and osteoblastic differentiation and their activity.^(^ ^16^, ^17^ ^)^ Changes in bone turnover lead to further alteration of bone quality and a consequent increase in bone fragility.^(^ ^2^ ^)^ Studies show that in a largely non‐obese population of T2D individuals, there is a suppression in bone turnover denoted by lower levels of Osterix^+^ and Osteocalcin^+^, without any significant change in BMD.^(^ ^20^ ^)^ Thus, the MKR model could provide vital mechanistic information on the impact of T2D as well as increased oxidative stress on T2D‐associated bone fragility.

Reactive oxygen species (ROS), formed under increased oxidative stress attributed to hyperglycemia, can enhance non‐enzymatic glycation (NEG) and lead to the formation and accumulation of AGEs.^(^ ^21^ ^)^ Pentosidine (PEN), one of the most studied AGEs, crosslinks collagen and is present in diabetic bone.^(^ ^22^ ^)^ PEN has become the gold standard to determine skeletal AGE accumulation. Conversely, carboxymethyl‐lysine (CML), a more robust AGE, has recently been shown to accumulate in bone and is present in greater concentrations than PEN.^(^ ^23^, ^24^ ^)^ Unlike PEN, CML is present as a side chain where its negatively charged carboxyl group can attract positively charged calcium ions promoting formation and growth of hydroxyapatite (HA), observed in T2D.^(^ ^25^, ^26^ ^)^ Furthermore, due to this chemical stoichiometry, CML may form a physical link between collagen and mineral,^(^ ^26^ ^)^ particularly in the interfibrillar space, where larger HA crystals grow. Consequently, CML may predict T2D bone fragility and explain why BMD consistently underpredicts T2D fractures.

In this study, we addressed several of the above questions regarding impaired bone quality with T2D using the non‐obese MKR model. We hypothesized that obesity independent T2D in the MKR model would display changes in bone structure seen in the human condition during non‐obese T2D. Additionally, MKR mice will display a decrease in bone material quality through changes in both the mineral and organic phases of the bone matrix resulting from glycation and glyco‐oxidative processes under hyperglycemia and increased oxidative stress. Furthermore, we posited that alterations in mineral and organic phases will display associations with impaired fracture properties and provide an explanation for increased T2D bone fragility.

## Materials and Methods

### Non‐obese mouse model of T2D

The non‐obese T2D condition in the MKR mouse model was developed by expression of the dominant negative human insulin‐like growth factor‐1 receptor (IGF‐1R) in the skeletal muscle under the muscle creatine kinase promoter^(^ ^27^ ^)^ on a genetic background resistant to diet‐induced obesity (FVB/N).^(^ ^28^ ^)^ Homozygous male MKR mice and FVB/N wild‐type (WT) controls were bred as described.^(^ ^13^ ^)^ The animals were housed within a full‐barrier facility on a 12‐hour light/dark cycle with full access to regular chow diet (PicoLab Rodent Diet, product number 5053; LabDiet, Brentwood, MO, USA). Although the progression of the T2D condition from insulin resistance to hyperinsulinemia (3–4 weeks after birth) and hyperglycemia (8 weeks after birth) has been detailed,^(^ ^27^ ^)^ body mass and nonfasting blood glucose levels were taken 8 weeks after birth to corroborate the diabetic condition (blood glucose >250 mg/dL) and the non‐obese state (body mass at 8 weeks after birth for FVB/N mice: 29.56 ± 1.50 g). At 15 weeks of age, all animals were euthanized. To remove the effect of laterality from the study, a left or right femur was randomly selected and extracted from each animal (*n* = 12/group), wrapped in saline‐soaked gauze and stored in Eppendorf tubes at −80°C until testing. All animal experiments were performed in compliance with the current standards specified in the Guide of the Care and Use of Laboratory Animals provided by the Association for Assessment and Accreditation of Laboratory Animal Care (AAALAC) and approved by the Mount Sinai Institutional Animal Care and Use Committee.

### Micro–computed tomography

**Fig 1:**
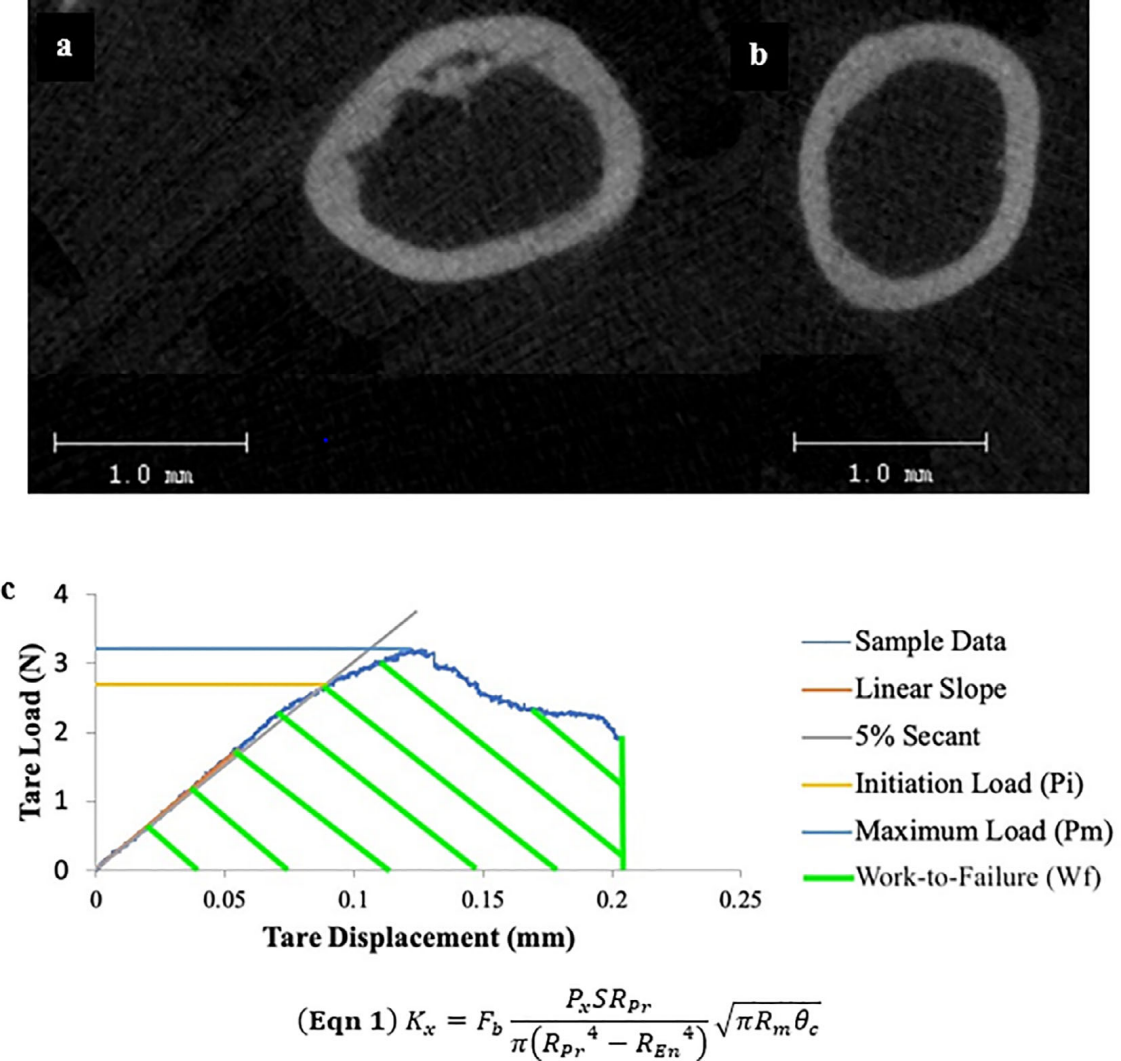
(*A*) Representative image from the WT group taken from the diaphyseal of the femora immediately proximal to the notch. (*B*) Similar figure from the MKR group. (*C*) Representative Load‐Displacement curve of notched three‐point bending fracture toughness testing. The initiation load (Pi; yellow line) was used to calculate the initiation toughness (Ki), determined from a sloped line at a slope of a 5% secant of the linear loading region. The maximum load (Pmax; light blue line) was used to calculate the maximum toughness (Kmax). The work‐to‐failure (Wf; green striped area) is the calculated as the area under the load‐displacement curve. Variables used to calculate toughness values: geometric parameter (Fb; derived from adjusted thickness to mean radii of each sample), S = span length; R_Per_ = periosteal radii; R_End_ = endosteal radii; R_mean_ = mean radii (R_mean_ = (R_Per_ + R_End_)/2) and θ_c_ = notch angle.^(^ ^31^ ^)^

All femurs were kept in Eppendorf tubes and submerged in saline during micro–computed tomography (μCT) scanning. Images were collected at 70 kVp, 114 mA, 200 ms integration time at resolution using a 10.5 μm voxel size (VivaCT40; Scanco Medical AG, Bassersdorf, Switzerland). The cross‐sectional geometry of the cortical bone was determined by a mid‐diaphyseal evaluation script over a defined volume of interest (VOI) including the mid‐point of the shaft and extending 400 μm proximally.^(^ ^29^, ^30^ ^)^ The cortical thickness (Ct.Th, mm), cortical area (Ct.Ar, mm^2^), total area (Tt.Ar, mm^2^), moment of inertia (I_xx_, mm^4^), periosteal and endosteal radii (Per.Rd and End.Rd, mm), and BMD (mg HA/cm^3^) were evaluated from the three‐dimensional (3D) reconstruction (Fig. 1*A*,*B*). BMD was derived from the average grayscale values of bone within the VOI. Gaussian filtration (σ = 0.8, midshaft support = 1), along with calibration constants for HA, was applied to the axial slices and the segmented cortical bone using a global threshold of 656 mg HA/cm^3^.

### Mechanical characterization of the matrix properties

Mechanical testing was performed to determine material properties associated with non‐obese T2D. In contrast to the previous mechanical testing conducted on the MKR model,^(^ ^13^ ^)^ we performed fracture toughness testing to minimize the effect of structure when comparing the quality of the bone matrix. To this end, a mid‐diaphyseal notch was placed on the anterior of the femur through the cortical wall to guide the point of crack initiation.^(^ ^32^ ^)^ The femoral head and condyles were removed using a IsoMet 1000 Precision Cutter (Buehler, Lake Bluff, IL, USA) and a diamond‐tipped saw blade to generate a reasonable facsimile of a hollow cylinder.^(^ ^31^ ^)^ The samples were then loaded to failure in a displacement‐controlled three‐point bending setting (Elf 3200; EnduraTEC, New Castle, DE, USA) using a ramp speed of 0.001 mm/s.^(^ ^30^, ^31^ ^)^ The distance between the lower fixtures of the three‐point bending apparatus that supported the sample's position, hereafter referred to as the *span length*, was held at 80% of the sample's length.^(^ ^33^ ^)^ The load–displacement data were used to calculate the initiation toughness, the critical stress intensity factor required to initiate the formation of a crack (see Eq. 1 in Fig. 1*C*, K_i_, MPa⋅m^1/2^)^(^ ^31^ ^)^; maximum toughness, a metric of the change in stress intensity at crack tip as it propagates under the largest sustained load (see Eq. 1 in Fig. 1*C*, K_max_, MPa⋅m^1/2^); and work‐to‐failure (W_f_, MNmm), representing the energy required to induce catastrophic failure.^(^ ^34^ ^)^ The difference between K_max_ and K_i_ was used to isolate the change in fracture toughness resisting the propagation of the crack after initial formation until the greatest load was reached, henceforth referred to as the *toughening effect* (ΔK, MPa⋅m^1/2^).^(^ ^35^ ^)^ The *cracking toughness* (K_cracking_, MJ/m^3^) was taken from the W_f_ normalized by the span length, which was then divided by the area resisting the load,^(^ ^34^, ^36^ ^)^ a metric characterizing the toughness of bone associated with transition to catastrophic crack growth such as one encountered during a fall.

### “In bulk” biochemical quantification of fluorescent AGEs

Quantification of fluorescent AGEs (fAGEs) “in bulk” is of importance to this study as their formation is driven by an array of glycation‐based processes.^(^ ^37^, ^38^, ^39^ ^)^ Measurement of fAGEs were performed on ~10‐mg sections of cortical femurs. The sections were collected from the area adjacent to the fracture surface extending proximally. The samples were lyophilized for 12 hours and then hydrolyzed in 6 N HCl for 16 hours at 110°C. After each hydrolysate dilution, fAGEs were measured using the fluorescence of quinine sulfate as a standard, described elsewhere.^(^ ^40^, ^41^, ^42^ ^)^ Briefly, samples were plated in triplicate (360 nm excitation and 460 nm emission) and read using a spectrophotometer (TECAN Nanoquant Infinite 200 Pro; Mannedorf, Zurich, Switzerland). Collagen content was calculated from the known volume of L‐hydroxyproline, which comprises approximately 14% of type I collagen. The fluorescence of each sample hydrolysate was normalized to the collagen content, that was determined using colorimetric assay with L‐hydroxyproline as a standard (570 nm absorbance). Values for fAGEs are reported as nanograms of quinine fluorescence per milligram of collagen (ng quinine fluorescence/mg collagen).

### Small‐angle X‐ray scattering

**Fig 2:**
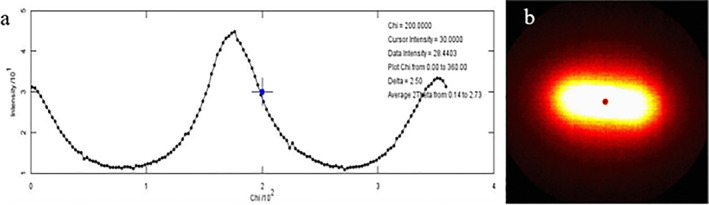
(*A*) Representative I‐versus‐X spectra used to extract the crystal thickness and orientation. (*B*) The corresponding heat map for the representative 2D spectra from which the intensity is extracted.

Random samples were selected between MKR and WT controls (*n* = 4/group). The cortical tissue samples were taken from the area immediately proximal of the fracture induced through mechanical testing. The samples were embedded in epoxy resin (AstroChem 1119; AstroChemical, Ballston Lake, NY, USA) and longitudinally sectioned to uniform thickness (300 μm). Two‐dimensional small‐angle X‐ray scattering (SAXS) spectra were acquired using a Bruker Nanostar‐U (Bruker, Bassersdorf, Switzerland) equipped with a Hi‐STAR two‐dimensional (2D) detector with real‐time photon counting capability (Bruker). The selection of locations for spectra acquisition (time/scan = 60 minutes; *n* = 3/sample; 50 kV; 24 mA) was done by nanoscale mapping. By creating a composition of 1‐second acquisitions, spaced apart by the beam width (0.4 mm), representative locations could be determined through the inspection of specimen's transmittance and cortex in comparison to the known standards for bone.^(^ ^43^ ^)^ Transmission coefficients were calculated for each point and used to attenuate the scattered intensities by subtracting the transmission coefficient of the epoxy medium. The spherical intensities of the scattering vector (Fig. 2 *B*) are dominated by the contribution of mineral due to its greater electron density compared to the organic matrix.^(^ ^44^ ^)^ The scattering vector (q) is a function of wavelength of the X‐rays (λ = 0.154 nm) and the angle between the X‐rays and the 2D detector (θ) determined as: q = 4sin(θ)/λ.^(^ ^43^, ^45^ ^)^ The intensity of the scattered electrons (I(q)) was evaluated over the full range of azimuthal angles (0 < ψ < 2π). In accordance with Porod's law (P = Iq^4^), Porod's constant was derived from the regions where Iq^4^ was constant (Porod regime). The mineral thickness (T) is described as the surface (S) to volume (V) ratio of the hydroxyapatite crystals (T = 4 V/S); mineral thickness calculations were performed in accordance with Porod's law by integrating the area of the Kratky plots (q^2^I versus q), yielding an invariant parameter (J), which was used to compute thickness (T = 4 J/πP) (Fig. 2 *A*). The in‐plane crystal orientation (ρ) was computed from the I(ψ) versus ψ plotted over the full azimuthal range.^(^ ^46^ ^)^

### Confocal Raman spectroscopy

**Fig 3:**
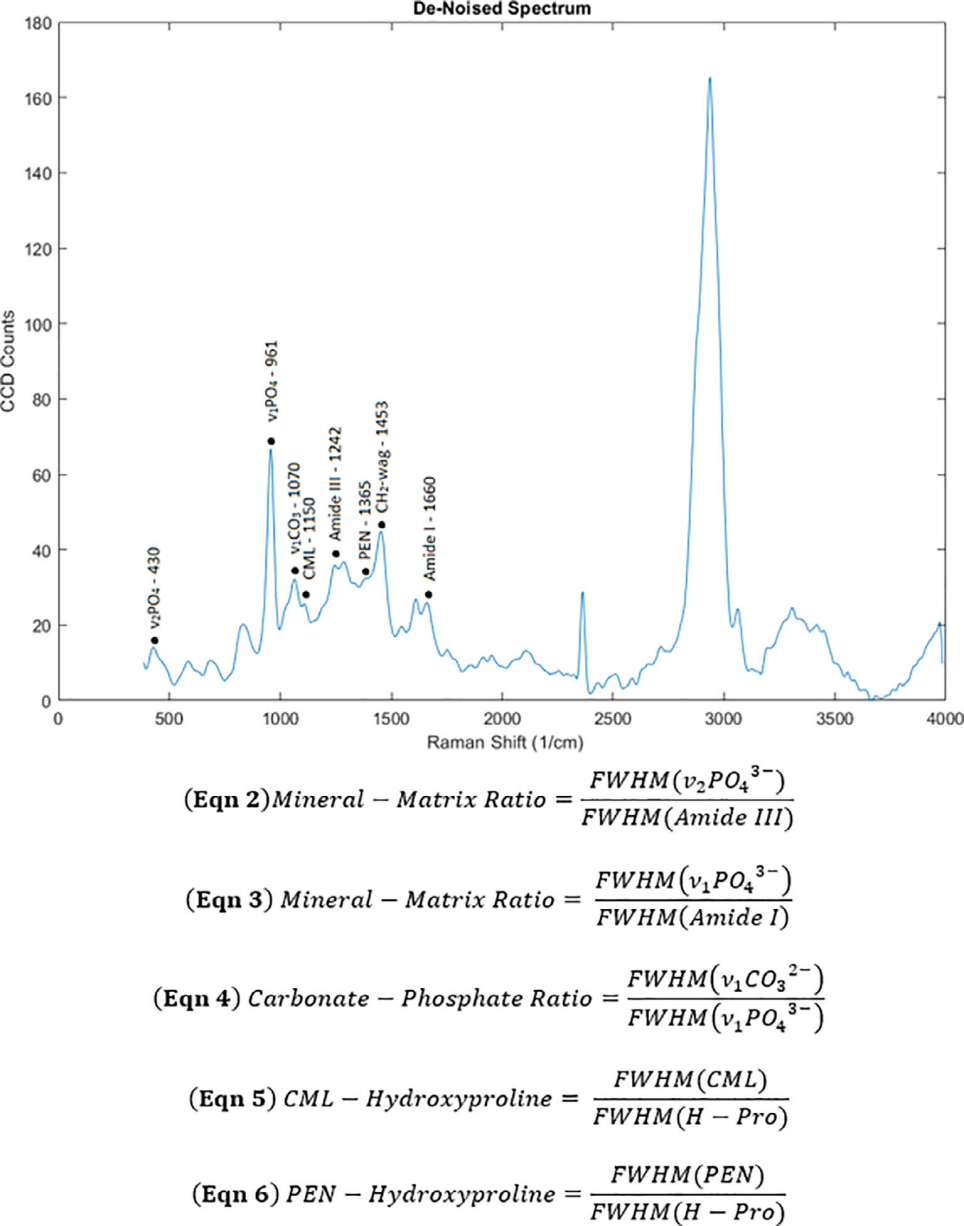
Representative plot of Raman intensity labeled with peaks used for analysis. The FWHM of the following peaks at the labeled Raman shifts (cm^−1^), correspond to skeletal extracellular matrix constituents: 430 (v_2_PO_4_^3−^), 961 (v_1_PO_4_^3−^), 1070 (v_1_CO_3_^2−^), 1150 (CML), 1242 (Amide III), 1365 (PEN), 1453 (CH_2_‐wag), and 1667 (Amide I).^(^ ^48^, ^54^ ^)^ FWHM = full‐width half‐maximum.

Confocal Raman spectroscopy is used to analyze the composition of bone's surface properties by generating spectra of relative intensities filtered from laser excitation.^(^ ^47^ ^)^ Confocal Raman spectroscopy was conducted on the same samples that were subjected to SAXS. The Raman signal was processed by an ultra‐high throughput spectrometer. Spectra were obtain using an excitation wavelength of 532 nm. Processing was done using a WITec Alpha 300R confocal Raman imaging system (WITec, Ulm, Germany) according to described protocols for Raman analysis.^(^ ^48^, ^49^ ^)^ The overall spectral resolution was 2 Δcm^−1^ at a spectral center of 2302 cm^−1^. Consistent with prior work, a single line spectrum was acquired over a longitudinal distance of 10 mm, originating distal to the fracture site with 20 accumulations at an integration time of 4 seconds from the periosteal surface of the sample.^(^ ^50^ ^)^ Cosmic ray reduction and background subtraction were applied to unfiltered spectra. Line acquisitions were averaged and convoluted by a moving point average pseudo‐Gaussian digital filter in order to create a combined spectrum for analysis using a custom MATLAB script (R2019b; MathWorks, Natick, MA, USA). The script was used to calculate the full‐width half‐maximum (FWHM) of the phosphate (ν_1_PO_4_^3−^ and ν_2_PO_4_^3−^), carbonate (ν_1_CO_3_^2−^), Amide I, Amide III (note that Amide III is less polarization dependent than other Amide bands^(^ ^51^ ^)^), methylene‐wag (CH_2_‐wag), PEN, and CML peaks based on the Raman shift (Fig. 3). Both PEN and CML are formed under glyco‐oxidative processes and these processes are known to be elevated in diabetic patients.^(^ ^39^, ^52^, ^53^, ^54^ ^)^ Verification of the CML peak location was done using a CML standard (Chem Impex International, Wood Dale, IL, USA) (Supplemental Fig. S1). The inverse of the ν_1_PO_4_^3−^ peak serves as a relative metric of bone crystallinity; the FWHM of the 957 cm^−1^ phosphate peak is inversely proportional to the mineral's *c*‐axis length.^(^ ^48^, ^55^, ^56^ ^)^ The relative ratio of the ν_2_PO_4_^3−^ to the Amide III (see Eq. 2 in Fig. 3) and the v_1_PO_4_^3−^ to the Amide I peak yielded the mineral‐to‐matrix ratio (See Eq. 3 in Fig. 3). The accumulation of Type‐B carbonate substitutions within the organic matrix was expressed by the relative ratio of ν_1_CO_3_^2−^ to ν_1_PO_4_^3−^ (See Eq. 4 in Fig. 3). As methylene (CH_2_‐wag) is ubiquitous across the organic matrix, the amount of CML and PEN was normalized to it, consistent with other confocal Raman spectroscopy results reported for these glyco‐oxidative products (see Eqs. 5 and 6 in Fig. 3).^(^ ^48^ ^)^ Subtraction of epoxy background revealed no significant impact of the peaks of interest (Supplemental Fig. S2). All values derived from spectroscopy were taken using consistent methodology and scale and, as such, used to determine the relative difference between the groups when compared statistically.

### Statistical analyses

All statistical analyses were performed using SPSS version 25.0 (IBM Corp, Armonk, NY, USA). As the sample properties were non‐normally distributed, determined using Kolmogorov‐Smirnov *Z*‐score analysis, nonparametric Mann‐Whitney *U* tests were performed with a significance level of 0.05. Spearman's ρ (α = 0.05) were used to determine the correlation coefficients between the variables reported. Correlations were first done within each group and then evaluated for the combined group. Fluorescent AGEs and mechanical testing were conducted on all samples, while SAXS and confocal Raman spectroscopy were performed on selected samples from each group (*n* = 4). Power analysis was conducted to assess the significance of the sample size. Correlations are reported for the combined group when no significant difference between the slope and intercept of the best fit line were found between an individual group and the combined group, as determined by analysis of covariance (ANCOVA). Variables with strong correlations were subsequently evaluated by stepwise linear regression to determine the most significant predictor of mineralization and fracture properties.

## Results

**Fig 4:**
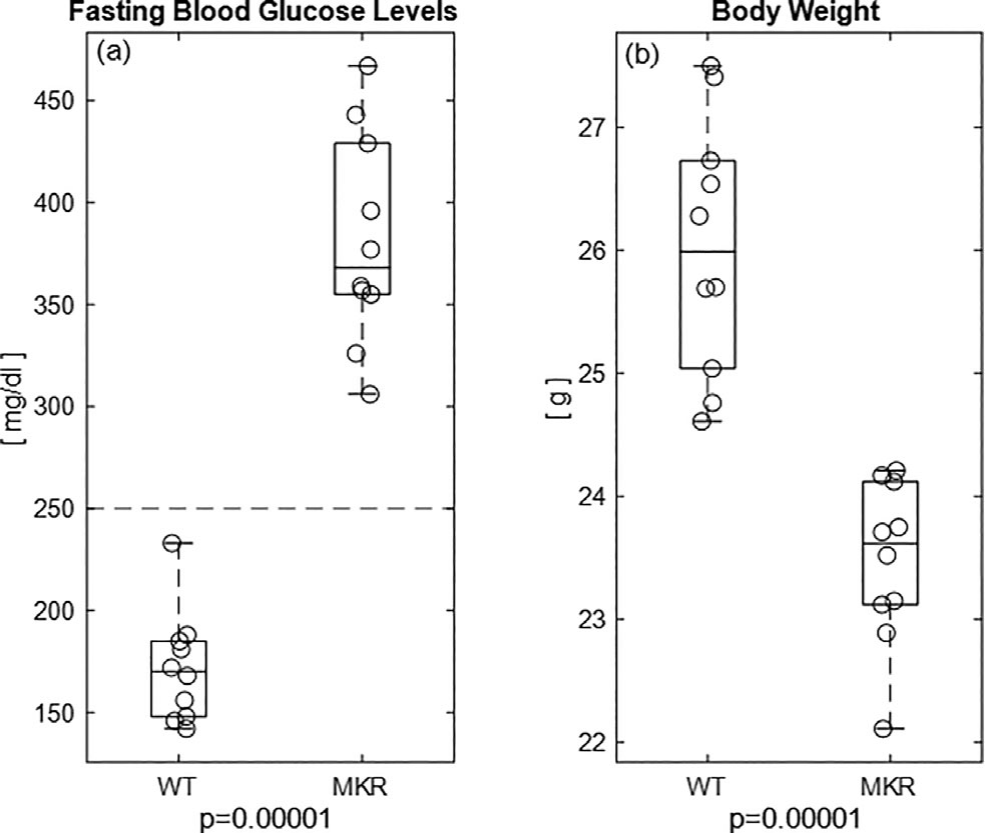
(*A*) By 8 weeks, the diabetic MKR group achieved diabetic levels of hyperglycemia (>250 mg/dL; dotted line^(^ ^93^ ^)^) while the WT group remained well below the blood glucose level for the diabetic state. (*B*) There is significant change in the body weight between the diabetic MKR mice compared to the WT controls.

By 8 weeks, all MKR mice progressed beyond the blood glucose threshold for the diabetic condition (percent difference [Δ] = 75.7%; Fig. 4*A*; blood glucoses level > 250 mg/dL; *p* = 0.0001). Conversely, none of the WT littermates reached the diabetic threshold by the same time point. Concurrently, the MKR mice also exhibited a 10% lower body mass compared to the WT controls (Δ10.3%; Fig. 4*B*; *p* = 0.0001).

**Fig 5:**
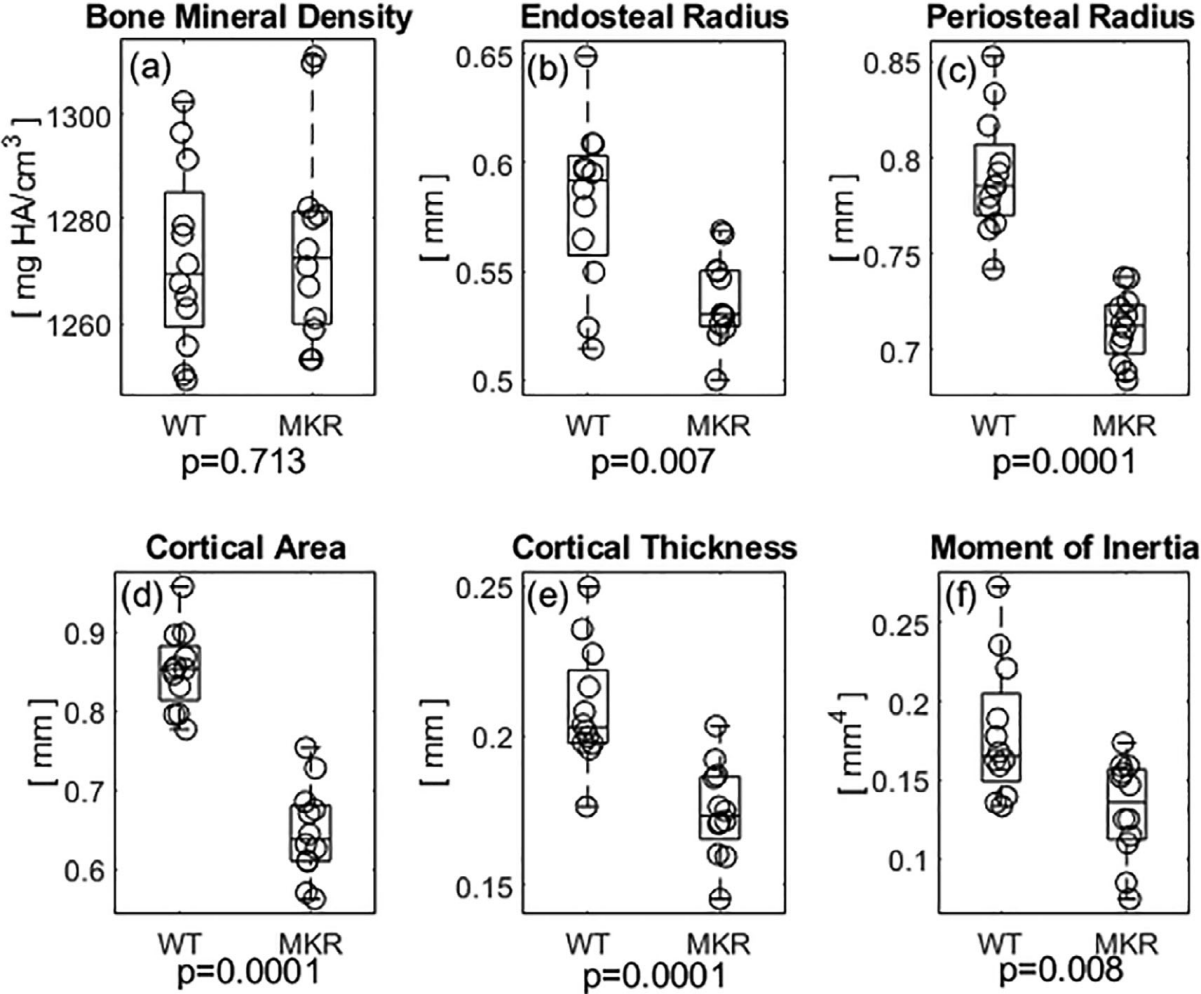
(*A*) The diabetic MKR exhibited slightly higher BMD values compared to WT mice although not statistically significantly so. (*B*) The average endosteal radius of the MKR femurs was decreased compared to the WT controls. (*C*) The average periosteal radius was significantly smaller in the MKR femurs compared to the WT controls. (*D*) The area of cortical bone was lesser in the MKR mice compared to the WT controls. (*E*) The thickness of the mid‐diaphyseal cortex was also significantly decreased in the MKR compared to WT controls. (*F*) Bone mass was less radially distributed in the MKR mice compared to the WT controls.

μCT revealed that BMD (Δ0.2%; Fig. 5*A*; *p* = 0.713) was not significantly different between MKR mice and WT controls. MKR mice had a smaller endosteal (Δ8.0%; Fig. 5*B*; *p* = 0.007) and periosteal diameter (Δ10.5%; Fig. 5*C*; *p* = 0.0001) compared to WT controls. The cortical area (Δ27.3%; Fig. 5*D*; *p =* 0.0001) and thickness of the cortex (Δ17.9%; Fig. 5*E*; *p* = 0.0001) were significantly reduced in the MKR group compared to the WT group. Additionally, bone mass was less radially distributed (lowered moment of inertia) in the MKR group, a marker of resistance to load in bending, compared to the WT group (Δ30.8%; Fig. 5*F*; *p* = 0.008).

**Fig 6:**
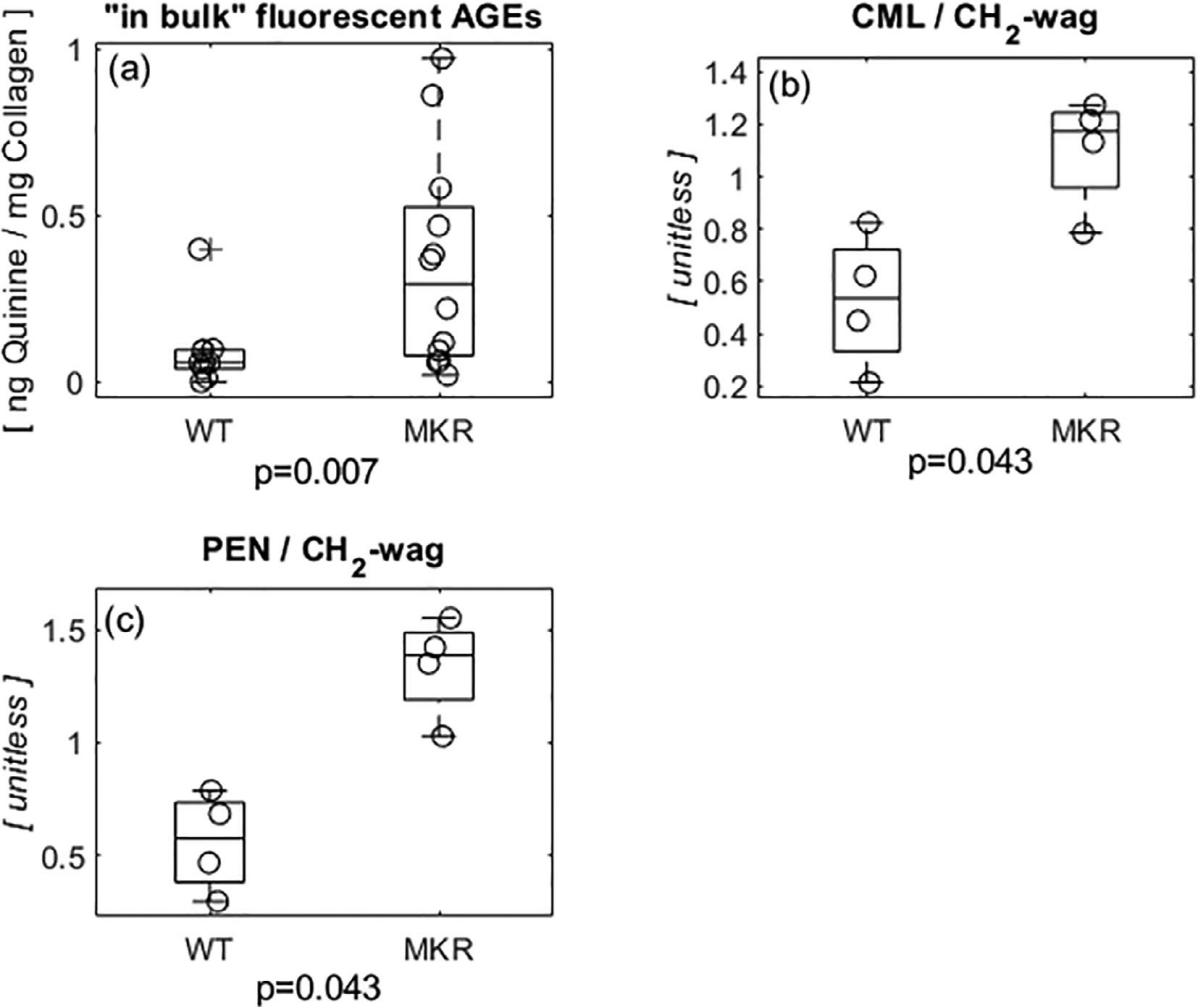
(*A*) Fluorescent AGEs, a product of non‐enzymatic glycation, accumulated nearly five times more in the diabetic group when compared to the WT controls. (*B*) CML, a product of glycoxidation normalized to the methylene peak (representative of type I collagen content), experienced significantly more accumulation in the diabetic MKR mice compared to the WT controls. (*C*) PEN, a fraction of the fluorescent AGEs present in bone, normalized to methylene (CH_2_‐wag) accumulated at nearly double the amount of seen in the WT controls. CML = carboxymethyl‐lysine; PEN = pentosidine.

Measurement of fAGEs revealed a fivefold greater accumulation in the MKR group compared to WT controls (Δ116.5%; Fig. 6*A*; *p* = 0.007). Normalized to methylene (CH_2_‐wag), the MKR group exhibited significantly greater formation of the carboxymethyl‐lysine (Δ70.4%; Fig. 6*B*; *p* = 0.043) and pentosidine (Δ82.2%; Fig. 6*C*; *p* = 0.043) when compared to relative levels in the WT group.

**Fig 7:**
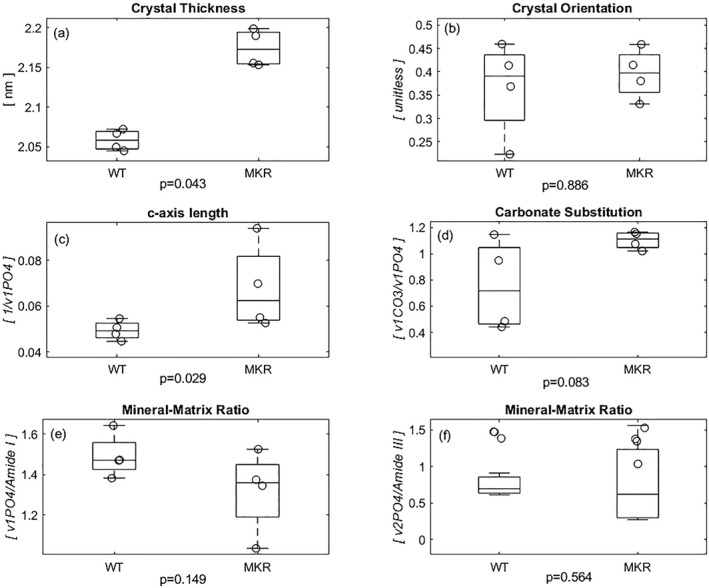
(*A*) The hydroxyapatite crystals within the MKR femurs' collagen fibrils were significantly thicker than those in the WT group. (*B*) Because crystal orientation ranges from 0 to 1, both the crystals in the diabetic and WT controls (ψ < 0.5) indicate orientation parallel to the axis of the bone, though not significantly different from each other. (*C*) The diabetic MKR group exhibited a significantly increased *c*‐axis length, which stems from its inverse relationship with the relative phosphate content of the hydroxyapatite crystals. (*D*) In addition to the increased growth, the MKR femora trended to exhibit more type‐B carbonate substitution within the inorganic when compared to the WT controls. (*E*) There was no difference in the relative amount of mineral determined by Raman peak of phosphate normalized to the amount of matrix determined by the Raman peak of Amide I. (*F*) There is no significant difference in the relative amount of mineral determined by Raman peak of less polarization‐dependent phosphate normalized to Amide III Raman peak representing the less polarization‐dependent matrix content. [Correction added on 15 February 2022, after first online publication: Figure 7 has been replaced]

Analysis at the nanoscale of the mineral phase demonstrated a significantly greater mineral crystal thickness in the MKR group (Δ5.5%; Fig. 7*A*; *p* = 0.043). There was no difference in the orientation of the HA crystals (Δ7.9%; Fig. 7*B*; *p* = 0.886). The relative increase in crystal growth, indicated by the thickness, was correlated (*p* < 0.001) with decreased relative phosphate content (which is inversely proportional to the *c*‐axis length^(^ ^48^ ^)^) in the MKR group (Δ31.5%; Fig. 7*C*; *p* = 0.029). There was a marginally greater presence of Type‐B carbonate substitution in MKR mice (Δ37.3%; Fig. 7*D*; *p* = 0.083).^(^ ^57^ ^)^ Although the above Raman spectroscopy analyses demonstrated altered mineralization in the MKR bones, the mineral‐to‐matrix ratio did not differ between groups (Δ12.2%, Δ8.1%; Fig. 7*E*,*F*; *p* = 0.149, *p* = 0.564). The comparison of the two groups is based on a power of 89.6%.

**Fig 8:**
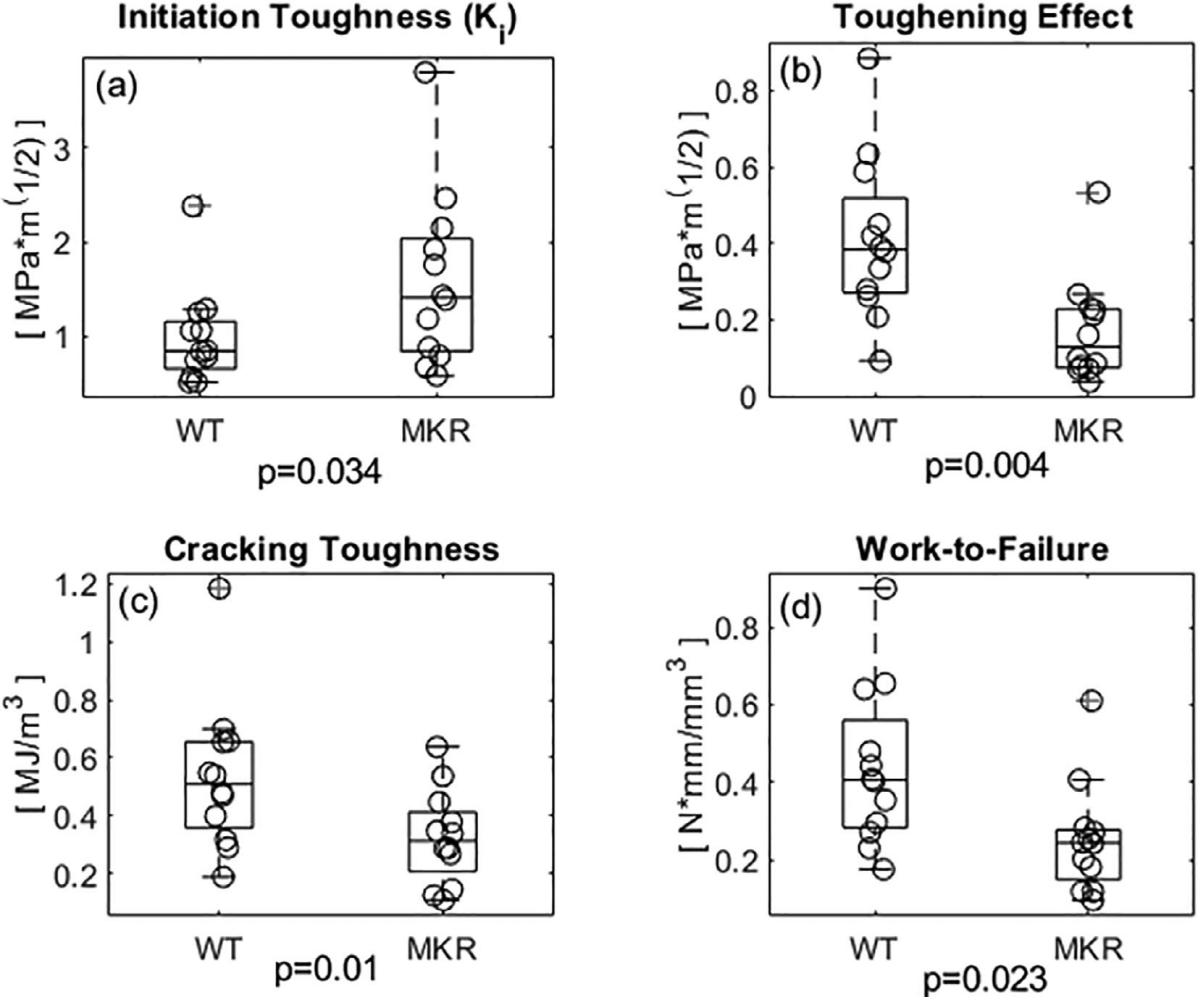
(*A*) The initiation toughness (K_i_), representative of the energy dissipation mechanisms imparted by the mineral component of bone's ECM, was significantly greater in the diabetic group compared to the WT controls. (*B*) The toughening effect (ΔK), the difference between the K_max_ and K_i_, another means of expressing the energy required to propagate a crack, is also reduced in the diabetic group. (*C*) The cracking toughness (K_cracking_) represents the energy required to propagate a crack through the bone and is significantly reduced in the diabetic group compared to the WT. (*D*) The work‐to‐failure (W_f_), which represents the work required to propagate a crack to failure normalized by the area resisting the load and the span length, was significantly reduced in the MKR group compared to the WT.

The MKR group exhibited significantly greater initiation toughness compared to the WT group (Δ46.7%; Fig. 8*A*; *p* = 0.034). Conversely, the MKR group had a nonsignificantly lower maximum toughness value when compared to WT controls (Δ22.1%; *p* = 0.235). The toughening effect (ΔK) was reduced in the MKR group (Δ81.3%; Fig. 8*B*; *p* = 0.004). The cracking toughness (K_cracking_) and work‐to‐failure (W_f_) were both decreased in the MKR group compared to WT controls (Δ48.6%, Δ53.5%; Fig. 8*C*,*D*; *p* = 0.010, *p* = 0.023).

**Fig 9:**
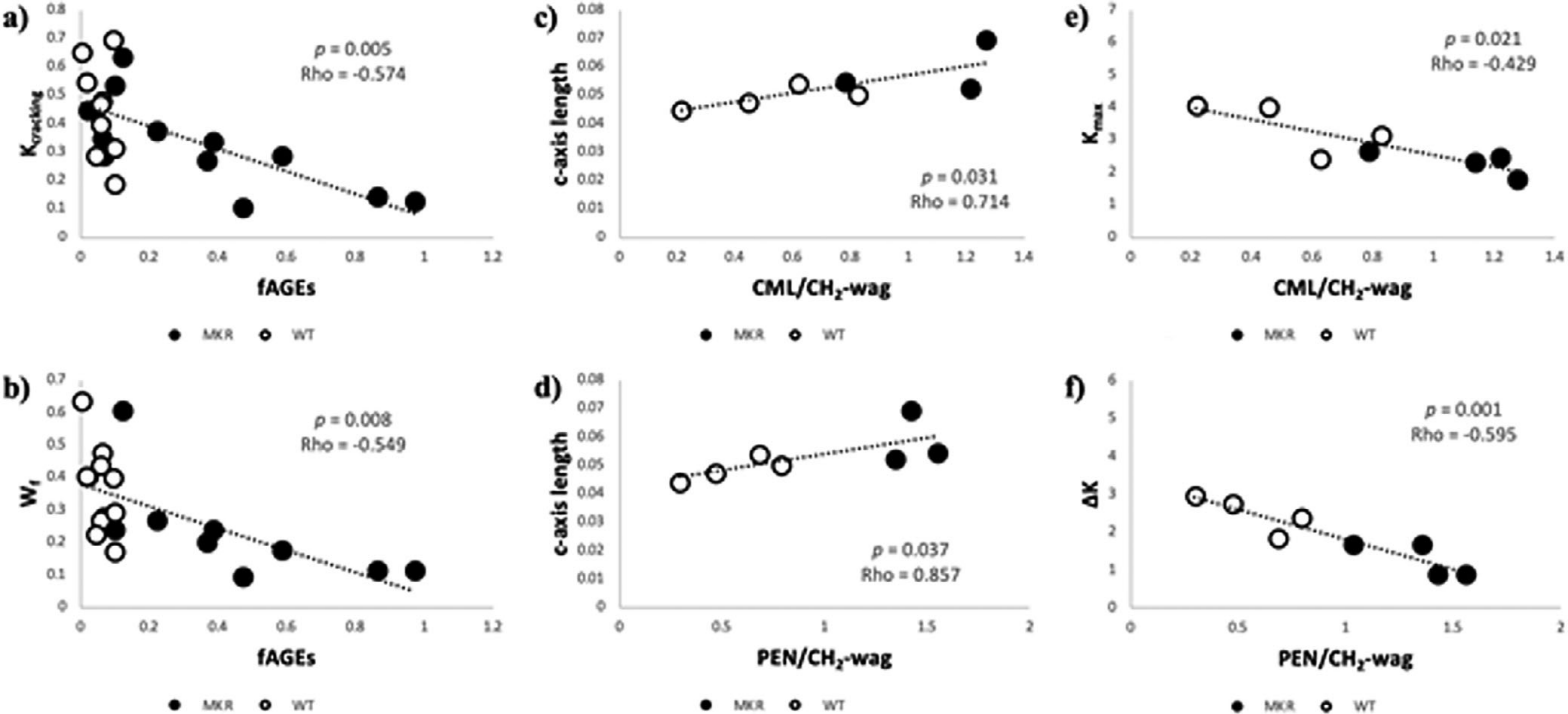
(*A*) Within the MKR group (black‐filled dots) there was a negative correlation between the cracking toughness and the accumulation of fAGEs, although these values do not correlate in the WT controls (black outlined, white filled dots). (*B*) There was a similar negative correlation between fAGEs and work‐to‐failure seen in the MKR group that was again absent in the WT controls. (*C*) The *c*‐axis length, a marker of bone mineral quality, had a positive correlation with CML/CH_2_‐wag between the groups. (*D*) The *c*‐axis length also had a positive correlation with PEN/CH_2_‐wag between the groups. (*E*) The maximum toughness had a negative correlation with CML/CH_2_‐wag which served a best predictor of maximum toughness. (*F*) The toughening effect had a negative correlation with PEN/CH_2_‐wag, which served as the best predictor of toughening effect (ΔK).

Associations between variables of interest are presented in Table 1. The cracking toughness and work‐to‐failure were negatively correlated with the accumulation of fAGEs (Fig. 9*A*,*B*; *p* = 0.005 and *p* = 0.008, respectively). The *c*‐axis length of the HA crystals was positively correlated with PEN and CML (Fig. 9*C*,*D*; *p* = 0.031 and *p* = 0.037, respectively). Stepwise linear regression revealed the most significant predictor of *c*‐axis length was CML/CH_2_‐wag content. Additionally, although both PEN/CH_2_‐wag and CML/CH_2_‐wag correlated negatively with maximum toughness, CML/CH_2_‐wag was the most significant predictor (Fig. 9*E*; *p* = 0.021). The toughening effect also had a strong negative correlation with CML and PEN content. Stepwise linear regression revealed that the toughening effect was more strongly predicted by PEN/CH_2_‐wag than CML/CH_2_‐wag (Fig. 9*F*; *p* = 0.001).

**Table 1:** Correlation of Mechanical Properties to Matrix Values

Property	BMD	Thickness	*ρ*	*c*‐axis	CO_3_/PO_4_	Min:Mat ratio	fAGE	CML/CH_2_	K_init_	K_max_	ΔK	K_cracking_	W_f_
Thickness	−0.238								−0.190	−0.881	−0.952	−0.524	−0.714
*ρ*	0.667.	0.119							−0.571	−0.405	−0.143	0.286	0.262
*c*‐axis	−0.250	0.929	0.179						−0.071	−0.893	−0.964	−0.286	−0.571
CO_3_/PO_4_	−0.667	0.619	0.024	0.607					−0.143	−0.548	−0.667	0.048	−0.095
Min:Mat ratio	0.000	−0.714	0.143	−0.571	−0.190				0.071	0.452	0.619	0.905	0.857
fAGE	−0.147	0.190	−0.310	0.179	0.429	−0.429			0.226	−0.054	−0.174	−0.574	−0.549
CML/CH_2_	−0.548	0.810	0.095	0.714	0.905	−0.381	0.190		−0.286	−0.786	−0.786	−0.071	−0.238
PEN/CH_2_	−0.381	0.833	0.048	0.857	0.786	−0.548	−0.651	0.762	0.143	−0.619	−0.929	−0.405	−0.571

Min:Mat ratio = mineral‐to‐matrix ratio.

## Discussion

In order to develop better therapeutic interventions, there is a need to identify the mechanisms through which the diabetic condition influences skeletal fragility. Because obesity and T2D are strongly linked comorbidities, we attempted to elucidate the mechanisms causing skeletal fragility by separating the impact of the two, thus removing the confounding influence of obesity on T2D. To address this issue, we used the MKR T2D mouse model to characterize obesity‐independent alterations in bone quality. Using a non‐obese T2D mouse model, here we determined the alterations in the mineral and organic components that are known to occur due to the presence of glycation and glyco‐oxidative processes under hyperglycemia and increased oxidative stress in T2D.^(^ ^26^ ^)^ Furthermore, we investigated the associations between the alterations of mineral and/or organic phases in bone matrix with impaired fracture properties in order to explain increased T2D bone fragility. Our study showed altered mineral crystal quality in T2D characterized by increased thickness and length despite no changes in BMD, and mineral‐to‐matrix ratio (ie, mineral quantity) as compared to controls. Our investigation of the organic matrix revealed accumulation of undesired posttranslational modifications of collagen, generated through glycation (eg, “in‐bulk” fAGEs) and glyco‐oxidation (eg, CML, PEN) processes.^(^ ^58^, ^59^, ^60^ ^)^ Increased AGE levels, along with the decreased periosteal and endosteal expansion suggest altered bone remodeling and turnover.^(^ ^61^, ^62^, ^63^ ^)^ As collagen and mineral (along with noncollagenous proteins and water) are known to contribute to material properties in bone,^(^ ^30^, ^64^, ^65^, ^66^ ^)^ we examined if the alterations to these components may impair and explain fracture resistance under the T2D condition.In this study, for the first time, we characterized the accumulation of AGEs in the organic matrix of the MKR mouse model in which T2D is independent of obesity. A similar magnitude of AGE accumulation was previously reported for obese rodent models of T2D.^(^ ^67^, ^68^ ^)^ However, our study reveals that glycation‐related modifications of organic matrix occur in the absence of obesity. Consistent with the progression of T2D in humans,^(^ ^69^ ^)^ we observed a significant decrease in the diameter of the periosteum and endosteum. We surmise that the decreased periosteal and endosteal expansion stems from decreased formation and resorption, respectively.^(^ ^70^ ^)^ Furthermore, the suggestion of diminished bone formation and resorption in MKR model are also consistent with the impaired osteoclastic and osteoblastic activity seen in and possibly associated with presence of increased AGE accumulation.^(^ ^61^, ^63^, ^71^, ^72^, ^73^, ^74^ ^)^

Of note, the reduction of skeletal muscle function derives from mitochondrial dysfunction known to occur in the MKR model^(^ ^75^ ^)^ and is consistent with the human T2D condition.^(^ ^76^ ^)^ Impaired mitochondrial oxidative capacity can lead to impaired energy metabolism as well as increased ROS production through impaired electron transport.^(^ ^77^ ^)^ Here, we observe that the increased accumulation of glyco‐oxidative AGEs, such as PEN and CML, which occurs in both serum and bone of T2D animal models and aging human bone, is mirrored by the non‐obese T2D condition. The significant accrual of both crosslinking (eg, PEN) and non‐crosslinking (eg, CML) AGEs in the non‐obese T2D model reveals a greater degree of organic matrix modification under the non‐obese T2D condition. Interestingly, despite the compromised supply of glucose to the skeletal muscle and the likely impaired loading, no reduction in BMD was observed in the diabetic bones.

The organic phase of bone governs the quality of mineral formation and maturation. The decreased rate of resorption with increased AGEs^(^ ^61^, ^78^ ^)^ provides an environment for the gradual maturation of hydroxyapatite crystals, as evidenced by in their increased thickness and elongation seen in the MKR model. This aspect of our study was notably different from a published study that used the KK/Ta + T2D mouse model displaying moderate obesity for which the authors observed decreased crystal thickness in alveolar bone,^(^ ^79^ ^)^ but not cortical bone as we present here. Recent work on T2D in humans (male and female donors) has revealed an increase in the mineral phase crystallinity, and this is consistent with the increased mineral thickness that we observed in the MKR mice.^(^ ^15^ ^)^ Interestingly, a different study that did not eliminated obesity as the confounding factor in diabetic humans, found no changes in crystallinity between male T2D donors compared to controls.^(^ ^14^ ^)^ Considering that our results show the difference observed in mineral crystallinity for the MKR mice males, we theorize that the differential growth of the mineral crystals between the obese and non‐obese condition can be caused by a combination of differential bone turnover,^(^ ^80^ ^)^ gender, and the differences between long and alveolar bone. Further work needs to be done to determine if these effects on mineral crystallinity can be considered as a therapeutic target in T2D.

The differential growth of HA mineral crystals in the non‐obese T2D mouse model used in our studies is supported by the increase in the amount of type‐B carbonate substitutions identified in the mineral phase. Type‐B carbonate substitutions, approximately parallel to the mineral's *c*‐axis, can deteriorate the lattice symmetry by introducing vacancies ultimately leading to impaired fracture resistance in the mineral.^(^ ^81^, ^82^ ^)^ Here, we show that CML serves as the most significant predictor of *c*‐axis length, a measure of mineral quality. Consequently, the prediction of maximum fracture toughness by CML may derive its basis from CML's association with the perturbed mineral quality. Taken together, the altered length, thickness, and composition of the mineral phase in the absence of any changes in mineral‐to‐matrix ratio suggest that the T2D condition affects mineral growth. In the absence of obesity, diabetic changes in the mineral phase of bone are derived from changes in bone mineral characteristics rather than BMD. More importantly, consistent with our proposal on the physical role for CML (see Introduction), the positive association between CML and mineral's *c*‐axis length support that negatively charged carboxyl group in CML may indeed attract positively charged calcium ions and promote formation of HA.^(^ ^25^, ^26^ ^)^ CML could therefore form a physical link to form between collagen and mineral, particularly in the interfibrillar space where larger HA crystals exist and grow.^(^ ^26^ ^)^ Such a link will explain bone fragility associated with increased CML formation.

The accumulation of alterations within the mineral and organic phases of the bone matrix impacts the intrinsic fracture properties. The initiation toughness characterizes crack initiation process at the yield point in bone (when plastic deformation begins).^(^ ^83^ ^)^ Bones with greater mineralization and lower collagen content would typically experience crack formation from a preexisting defect (such as notch in our experimental setup or a preexisting microcrack in vivo) at higher stresses.^(^ ^84^, ^85^ ^)^ Furthermore, the suggested elongation of the *c*‐axis length implied by increased crystallinity in the diabetic femurs may induce residual strains within the inorganic matrix.^(^ ^81^ ^)^ This pre‐strain may also impart initial resistance to crack formation but at the cost of creating a more brittle‐behaving material. As such, the elevation of initiation toughness observed in the MKR mice can be explained by the increased crystal growth seen in the diabetic bones.^(^ ^86^ ^)^ Furthermore, increased crystallinity, reported here in MKR model, has previously been associated by multiple logistic regression with cortical bone fragility in a non‐T2D human population.^(^ ^87^ ^)^ Our group (Poundarik and colleagues^(^ ^88^ ^)^) has also previously shown that increased collagen glycation does not affect initiation toughness, corroborating the impact of mineralization to increase resistance for crack initiation. Also, although water content throughout bone's hierarchical structure has been shown to impart resistance to fracture,^(^ ^66^, ^87^ ^)^ samples in this study were dehydrated for conducting other assays and bound water content could not be assessed. However, AGEs were investigated in detail and previous work has shown that they do account for loss of water.^(^ ^86^ ^)^

The significantly elevated levels of AGEs in the bone matrix, reported here, may alter various energy dissipating processes in bone and impair the fracture properties.^(^ ^24^, ^63^, ^88^, ^89^, ^90^, ^91^ ^)^ For example, PEN is known to crosslink the collagen molecules, and therefore stiffen the organic matrix.^(^ ^92^, ^93^ ^)^ And here, we show that accumulation of PEN predicts the loss of toughening effect in bone. PEN has also been associated with bone fragility^(^ ^94^, ^95^ ^)^ through reduced crack bridge formation^(^ ^96^ ^)^ that impairs energy dissipation and consequently the postyield fracture properties of bone. Impact of AGE accumulation on increased bone fragility is further supported by significant negative correlations between “in‐bulk” fAGEs to K_cracking_ and W_f_ (Fig. 9). Notably, cracking toughness represents the majority of energy dissipation mechanisms after the initial crack formation. Moreover, maximum toughness is predicted by CML (a non‐crosslinking AGE) which, as discussed above, may effectively function as a crosslink by providing a physical link between the organic and inorganic phases.^(^ ^75^ ^)^

Our study did not include obese T2D mice. Although inclusion of such a group would have enabled a direct comparison between the magnitude of matrix alterations between non‐obese to obesity‐enhanced T2D, the breadth of work detailing skeletal fragility in obese humans and murine models^(^ ^5^, ^6^, ^97^, ^98^ ^)^ as well as the difficulties associated with inducing obesity in an FVB/N mouse (used as control for MKR) would have made inclusion of an obese group a more limiting than beneficial factor.^(^ ^28^, ^99^ ^)^ The MKR model does indeed provide a unique and sufficient platform to interrogate the bone matrix alterations and their impact on bone fragility. In particular, it serves as clinically relevant model to assess diabetic complications/comorbidities through transgenic induction without the confounding nature of other methods of inducing non‐obese T2D, such as streptozotocin. The progression of diabetes in the MKR model mirrors the respective progression observed in humans displaying “normal” body mass index (BMI),^(^ ^100^ ^)^ and it also reflects the development of complications, such as dyslipidemia.^(^ ^27^ ^)^ Notably, dyslipidemia has been shown to contribute to cardiovascular disease^(^ ^101^ ^)^ and to the accumulation of AGEs in diabetic individuals.^(^ ^102^ ^)^ As such, although there is a large non‐obese T2D population of both men and women located in India and southeastern Asian,^(^ ^103^ ^)^ studies regarding the difference in fracture risk in humans in the absence of obesity remain unexplored regardless of the global region.

In summary, our study addressed the changes in the bone matrix, inherent to the diabetic condition, that explain the reduction in bone quality in the absence of obesity. Altered mineral quality, in addition to the increased accumulation of modifications within the organic phase of bone matrix, contributes to the impaired energy dissipation mechanisms and increased skeletal fragility. The impact of altered mineral quality in diabetic skeletal fragility and its contribution to enhancing our understanding of how T2D pathology affects bone quality cannot be understated.

## Conflict of Interest

All authors state that they have no conflicts of interest.

### PEER REVIEW

The peer review history for this article is available at https://publons.com/publon/10.1002/jbm4.10583.

## Supporting information


**Fig. S1**. A confocal Raman spectra of a CML standard of 750 ng/mL of H2O. The vertical green line at the reported center of the CML peak. While the peak is within the expected range for CML, the slight rightward shift compared to those reported in literature (over the same range^23^) may be caused to the difference in the spectral centers and the impact of H2O in our standard spectra.

**Fig. S2**. Upon performing a background subtraction of a pure epoxy spectra using confocal Raman spectroscopy and comparing it to the representative bone used for the scan. The percent difference between the two spectra was 8.77% with no significant difference in the FWHH of the of the peaks of interest.
