# Supplementary material for: Non‐Obese MKR Mouse Model of Type 2 Diabetes Reveals Skeletal Alterations in Mineralization and Material Properties
Source: JBMR Plus. 2021 Dec 16;6(2):e10583. doi: 10.1002/jbm4.10583 (PMC8861985; doi:10.1002/jbm4.10583)
Supplement: Supplementary file 1 — Fig. S1. A confocal Raman spectra of a CML standard of 750 ng/mL of H2O. The vertical green line at the reported center of the CML peak. While the peak is within the expected range for CML, the slight rightward shift compared to those reported in literature (over the same range23) may be caused to the difference in the spectral centers and the impact of H2O in our standard spectra. [file JBM4-6-e10583-s001.pdf]

Supporting Information #1

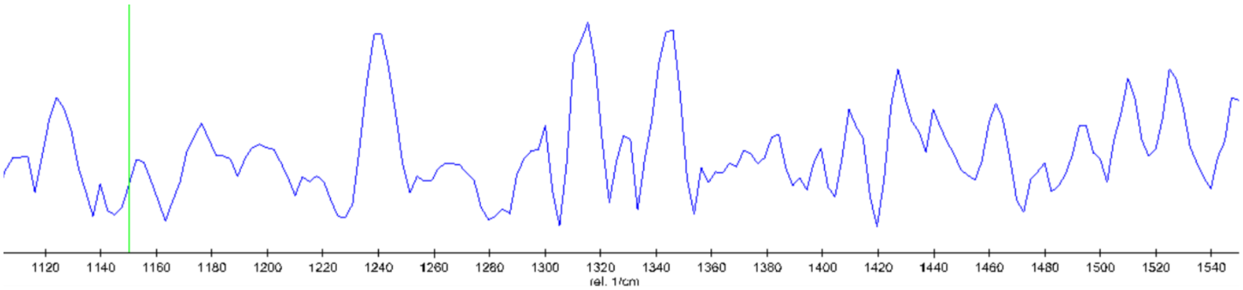

Figure Legend

Supplemental Figure 1. A confocal Raman spectra of a CML standard of 750 ng/mL of H_2O. The vertical green line at the reported center of the CML peak. While the peak is within the expected range for CML, the slight rightward shift compared to those reported in literature (over the same range[23]) may be caused to the difference in the spectral centers and the impact of H2O in our standard spectra.
