# Supplementary material for: Non‐Obese MKR Mouse Model of Type 2 Diabetes Reveals Skeletal Alterations in Mineralization and Material Properties
Source: JBMR Plus. 2021 Dec 16;6(2):e10583. doi: 10.1002/jbm4.10583 (PMC8861985; doi:10.1002/jbm4.10583)
Supplement: Supplementary file 2 — Fig. S2. Upon performing a background subtraction of a pure epoxy spectra using confocal Raman spectroscopy and comparing it to the representative bone used for the scan. The percent difference between the two spectra was 8.77% with no significant difference in the FWHH of the of the peaks of interest. [file JBM4-6-e10583-s002.pdf]

Supporting Information #2

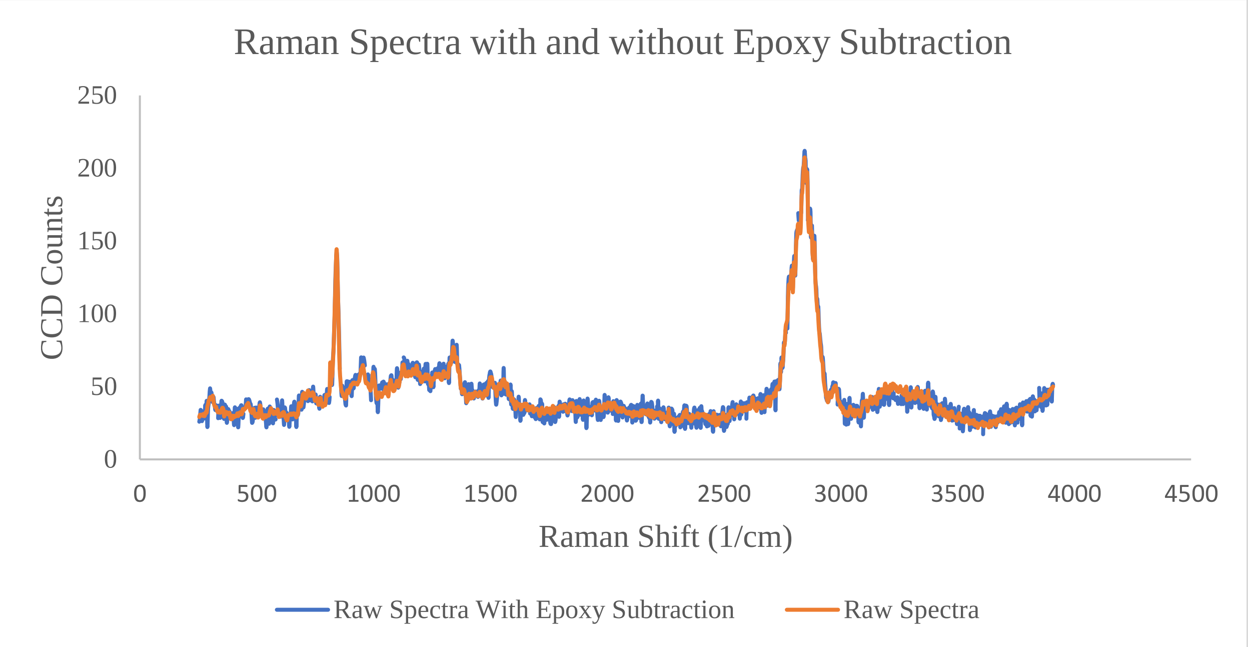

Figure Legend

Supplemental Figure 2. Upon performing a background subtraction of a pure epoxy spectra using confocal Raman spectroscopy and comparing it to the representative bone used for the scan. The percent difference between the two spectra was 8.77% with no significant difference in the FWHH of the of the peaks of interest.
